# The CYP2D6 phenotyping performance of single-point saliva metabolic ratio in a healthy Chinese Han population

**DOI:** 10.3389/fphar.2025.1438760

**Published:** 2025-02-21

**Authors:** Yongfa Huang, Zhangyuting He, Xiaoduo Guan, Pei Hu, Rui Chen

**Affiliations:** ^1^ Clinical Pharmacology Research Center, Peking Union Medical College Hospital, Chinese Academy of Medical Sciences and Peking Union Medical College, Beijing, China; ^2^ Liver Transplantation Center, National Clinical Research Center for Digestive Diseases, Beijing Friendship Hospital, Capital Medical University, Beijing, China; ^3^ Clinical Center for Pediatric Liver Transplantation, Capital Medical University, Beijing, China; ^4^ Department of Internal Medicine, Peking Union Medical College Hospital, Peking Union Medical College & Chinese Academy of Medical Sciences, Beijing, China

**Keywords:** CYP2D6, saliva metabolic ratio, activity score, non-invasive phenotyping, dextromethorphan

## Abstract

The CYP2D6 plays critical roles in drug metabolism, and its inter-individual variability should be properly addressed in clinical practice. Our research aimed to assess the performance of single-point saliva metabolic ratio (MR) in a healthy Chinese Han population using dextromethorphan as the probe drug. MR was determined as the ratio of parent drug to metabolite in 3 h saliva, 3 h plasma, and 0–3 h urine post-ingestion. 416 healthy volunteers were enrolled, with 290 (69.7%) male participants and a median body mass index (BMI) of 23.1 kg/m^2^. The Spearman correlation coefficients were 0.876 between plasma and saliva MR, and 0.746 between plasma and urine MR. The population was clustered into four metabolizer classes with either plasma or saliva MR, but into two metabolizer classes with urine MR. The saliva-based clustering agreed well with plasma-based clustering (Cohen’s Kappa 0.689). A clear negative correlation was observed between Activity Score (AS) and saliva MR, and linear regression revealed that overweight population had significantly lower saliva MR than others. In conclusion, single-point saliva MR performed better than urine MR with satisfactory correlation with plasma MR and effective separation of four metabolizer classes. Predicting saliva MR with the AS system was more accurate when BMI was considered.

## 1 Introduction

The highly polymorphic gene *CYP2D6* encodes the enzyme cytochrome P450 2D6, and the alleles can be classified into increased, normal, decreased, and no function groups according to the observed enzymatic activity (Caudle et al., 2020). Commonly found in microsomes and other cytoplasmic structures in hepatocytes, CYP2D6 functions as a monooxygenase and takes part in the biotransformation of numerous clinically used drugs (Puszkiel et al., 2021; Muriel et al., 2023). When probed with dextromethorphan (DM) or other substrates, the *CYP2D6* phenotype can be demonstrated with metabolic ratio (MR) and classified into poor (PM), intermediate (IM), normal (NM), or ultrarapid (UM) metabolizers accordingly (Gaedigk et al., 2008).

Determining the drug metabolism phenotype is of critical importance to select the proper medication and dose in clinical practice. Based on previously confirmed data, the Activity Score (AS) system was developed in order to deduce related drug metabolism phenotype based on *CYP2D6* genotyping information (Gaedigk et al., 2008). Though continually evolving, the latest version of AS system merely explained 40% of variability in DM metabolizing rates, and could not replace the direct phenotyping methods yet (Dalton et al., 2020; Hertz et al., 2015).

Traditional pharmacokinetic analysis requires a time series of plasma drug concentration to demonstrate the whole plasma concentration-time profile. For *CYP2D6* phenotyping specifically, our previous study has shown that single-point plasma MR from 1 to 30 h post-ingestion is a satisfactory substitute for traditional MR by area-under-curve method, but its application is still limited by the necessity of venous puncture (Chen et al., 2016). Another classical method, urine MR analysis, requires urine collection during a long period of time, which precludes urine method from routine clinical practice (Gaedigk et al., 2008). Also, urine MR can be influenced by urinary pH (Labbé et al., 2000), and is not applicable in anuric patients with renal failure (Hou et al., 1996).

Saliva is an easily accessible body fluid and a suitable specimen for drug concentration determination, and has been investigated as a proper substitute for plasma and urine since 1990s (Hou et al., 1996; Hou et al., 1991). Its convenience has drawn attention from clinicians, pharmacists, and pharmacologists, and the usefulness of saliva in anticonvulsant monitoring has been firmly established (Kim et al., 2020). For cytochrome P450 phenotyping, the time series of saliva drug concentration has been used in phenotyping of *CYP2D6*, *CYP3A*, and *CYP1A2* with satisfactory results up till now (Link et al., 2008; Perera et al., 2011; Chen et al., 2017; Jin et al., 2022). However, the usefulness of single-point saliva MR has not been fully investigated in a large enough representative healthy East Asian population. Our study aims to demonstrate the performance of single-point saliva MR in phenotyping, to compare it with plasma and urine counterparts, and to confirm its correlation with the AS system and other factors.

## 2 Materials and methods

### 2.1 Study subjects

This study was performed in accordance with the Guidelines for Good Clinical Practice and the Declaration of Helsinki. The research protocol was reviewed and approved by the Ethical Committee of Peking Union Medical College Hospital, Beijing, China. In total, 421 healthy unrelated subjects of Han people from Chinese mainland were voluntarily enrolled to clarify the CYP2D6 phenotyping performance of different body fluid samples, and written informed consent was obtained from each subject (Chen et al., 2017). Subjects had a detailed physical examination including routine urinalysis, complete blood count, comprehensive metabolic panel, and 12-lead electrocardiography in order to exclude the ones with any of the following situations: a history of hematologic, gastrointestinal, renal, or hepatic abnormalities; human immunodeficiency virus, syphilis, hepatitis C or B infection; allergy to dextromethorphan; other acute or chronic diseases that were not cured at recruitment. Consuming grapefruit juice, caffeinated beverages, or alcohol was not allowed in the last 24 h before DM administration, nor until all samples were collected. Subjects were also instructed to refrain from ingesting herbal remedies or medications for a minimum of 1 week before the study, and to refrain from smoking for a minimum of 3 days before the study. All the collected data were later introduced into regression analysis, and those subjects with items missing were excluded.

### 2.2 CYP2D6 genotyping and AS assignment

Whole genomic DNA was extracted from white blood cells in peripheral blood samples using a Wizard TM Genomic Purification Kit (Promega, United States), and then different alleles were detected with Sanger sequencing (Chen et al., 2017). To be concise, the genomic region including *CYP2D6* gene and its 5′ and 3′ untranslated regions (M33388 positions −2,182–4,482) was amplified. As the whole DNA segment flanking *CYP2D6* gene was too long for one single Sanger sequencing, six different primers evenly scattered across the gene were designed to perform six independent Sanger sequencing from the respective starting points. Since the six sequenced sub-segments overlapped with each other at the juncture, the single nucleotide polymorphisms (SNPs) of complete *CYP2D6* gene were determined successfully. After cleansing with the PCR purification Kit (Capitalbio, China), Sanger sequencing was performed with BigDye Terminator v3.1 Cycle Sequencing Kit (Applied Biosystems, United States) under the following program: 96°C/1 min for denaturing, 25 cycles of 96°C/10 s–50°C/5 s–60°C/4 s. The final reaction product was purified via ethanol/ammonium acetate precipitation, and then analyzed using the ABI 3730XL Genetic Analyzer (Applied Biosystems, United States) and Sequence Variation Analysis v1.2 (Capitalbio, China). Deletion (*5) as well as duplication were detected using a duplex long polymerase chain reaction-based method (DLPCR) originally developed by Løvlie et al. (1996) and Hersberger et al. (Hersberger et al., 2000), with minor modifications. Briefly, the *CYP2D6* flanking region changes due to deletion or duplication resulted in length variation of DNA clones amplified with elaborately designed primers, which was then visualized using 1.5% agarose gel electrophoresis. In case of duplication, the whole duplicated segment was specifically cloned and sequenced with the above Sanger method to determine which allele it was. The naming of alleles, genotypes, AS and metabolizer subgroups were determined according to SNPs from Sanger sequencing, deletion and duplication from DLPCR, latest allele scoring in Pharmacogenomics Knowledgebase (Whirl-Carrillo et al., 2021), and the 2020 consensus recommendations from the Clinical Pharmacogenetics Implementation Consortium and Dutch Pharmacogenetics Working Group (Caudle et al., 2020), which had been validated in our (Jin et al., 2022) and Wang et al. (2024) studies. Since the specific allele duplication number was not determined, all duplicated alleles were attributed with twice the original activity value as Gaedigk et al. originally reported when developing the AS system (Gaedigk et al., 2008): activity value = 2 for *1xN and *2xN, activity value = 0.5 for *10xN and *41xN (Duarte et al., 2024). The rare alleles with uncertain function, including *90, were assigned with 0 activity value. The potential bias from this manual assignment was limited according to the sensitivity analysis, in which the main findings were found unaffected.

### 2.3 CYP2D6 phenotyping with DM

Each subject was provided with 15 mg of DM (Tylenol Cold Tablet containing DM, Johnson and Johnson Investment Ltd., Shanghai, China), along with 300 mL water (Chen et al., 2017). Saliva (2–3 mL each) and peripheral venous blood samples (8–9 mL each) were collected at 3 h post-ingestion, while all urine were collected during the 0–3-hour interval post-ingestion, which was proved effective in a previous study by Chládek et al. (2000). Saliva samples were collected into test tubes directly from the oral cavity using sterile pipettes, and were then centrifuged at 4,000 rpm, 4°C for 15 min to separate the supernatants for further experiments. Concentrations of DM and unconjugated dextrorphan (DX) in all samples were determined with a sensitive and validated high performance liquid chromatography tandem mass spectrometry assay using established standards without hydrolysis (Hou et al., 1991; Hu et al., 1998). To be specific, DM and DX were detected using fluorescence detectors (Millipore, United States) at wavelengths of 200/310 nm (excitation/emission), and the lower limit of quantification was 0.05 ng/mL for both DM and DX in all samples. The MR of DM over DX was adopted to assess the CYP2D6 enzymatic activity in plasma, saliva, or urine samples.

### 2.4 Statistical analysis

Data were displayed as median (lower quantile, higher quantile). To enhance the readability of the results and the performance of statistical analyses, MR underwent log transformation (base 10) in part of the results. The strength of correlation was measured with Spearman’s correlation coefficient, and the agreement between different classification systems was measured with Cohen’s kappa coefficient. Model-based clustering by R package “mclust” was performed to divide log10 of MR into mixtures of univariate normal distributions using R commands formatted as “clusters < - Mclust (data, modelNames = “V”)”, and the related statistical principles are demonstrated in the book composed by Scrucca et al. (2016). Both-direction (forward and backward) stepwise linear regression was performed to investigate the impact of AS and other factors on MR, and variable inclusion/exclusion was determined with Akaike information criteria. All statistical analyses were performed with R statistics (version 4.2.0, https://www.R-project.org/).

## 3 Results

### 3.1 Demographic characteristics, CYP2D6 genotypes and AS

In total, 416 subjects with complete data record were included in the final analysis with 290 males and 126 females (Table 1). These subjects comprised a representative population, with a median age of 29 (Jukić et al., 2021; Krogstad et al., 2021) years and a median body mass index (BMI) of 23.1 (21.5, 24.8) kg/m^2^. Of all *CYP2D6* alleles, *10 was the most frequent allele (45.4%) in the study population, and the second and third most frequent ones were *1 (25.1%) and *2 (13.2%). Allele duplication was detected in 11 subjects, including 1 *1xN, 3 *2xN, 5 *10xN, and 2 *41xN. The top three most frequent genotypes were *1/*10 (22.4%), *10/*10 (20.4%), and *2/*10 (13.0%), respectively. Over four-fifths of all subjects (84.6%) were attributed with an AS less than 2, and the majority of participants were classified into NM (54.1%) and IM (43.5%). The *CYP2D6* genotype of all 416 participants was presented in Supplementary Table S1.

**TABLE 1:** Demographic characteristics, *CYP2D6* genotypes, and AS.

N	416
Age (years)	29 (25, 35)
Weight (kg)	64.2 (58.8, 70.5)
BMI (kg/m^2^)	23.1 (21.5, 24.8)
Male Gender	290 (69.7%)
*CYP2D6* allele frequencies^a^	
*1	209 (25.1%)
*1xN	1 (0.1%)
*2	110 (13.2%)
*2xN	3 (0.4%)
*4	7 (0.8%)
*5	74 (8.9%)
*10	378 (45.4%)
*10xN	5 (0.6%)
*14	6 (0.7%)
*35	1 (0.1%)
*41	28 (3.4%)
*41xN	2 (0.2%)
*49	5 (0.6%)
*52	1 (0.1%)
*90	1 (0.1%)
*94A	1 (0.1%)
*CYP2D6* genotype frequencies	
*1/*10	93 (22.4%)
*10/*10	85 (20.4%)
*2/*10	54 (13.0%)
*5/*10	35 (8.4%)
*1/*2	34 (8.2%)
*1/*1	22 (5.3%)
*1/*5	17 (4.1%)
*10/*41	16 (3.8%)
Others	60 (14.4%)
AS =	
0	6 (1.4%)
0.25	41 (9.9%)
0.5	101 (24.3%)
0.75	8 (1.9%)
1.0	31 (7.5%)
1.25	155 (37.3%)
1.5	10 (2.4%)
2.0	60 (14.4%)
3.0	4 (1.0%)
Metabolizer class by AS	
PM	6 (1.4%)
IM	181 (43.5%)
NM	225 (54.1%)
UM	4 (1.0%)

BMI: body mass index.

^a^ The copy number variant was treated as a separate allele here.

### 3.2 Comparing saliva, plasma, and urine MR

**FIGURE 1:**
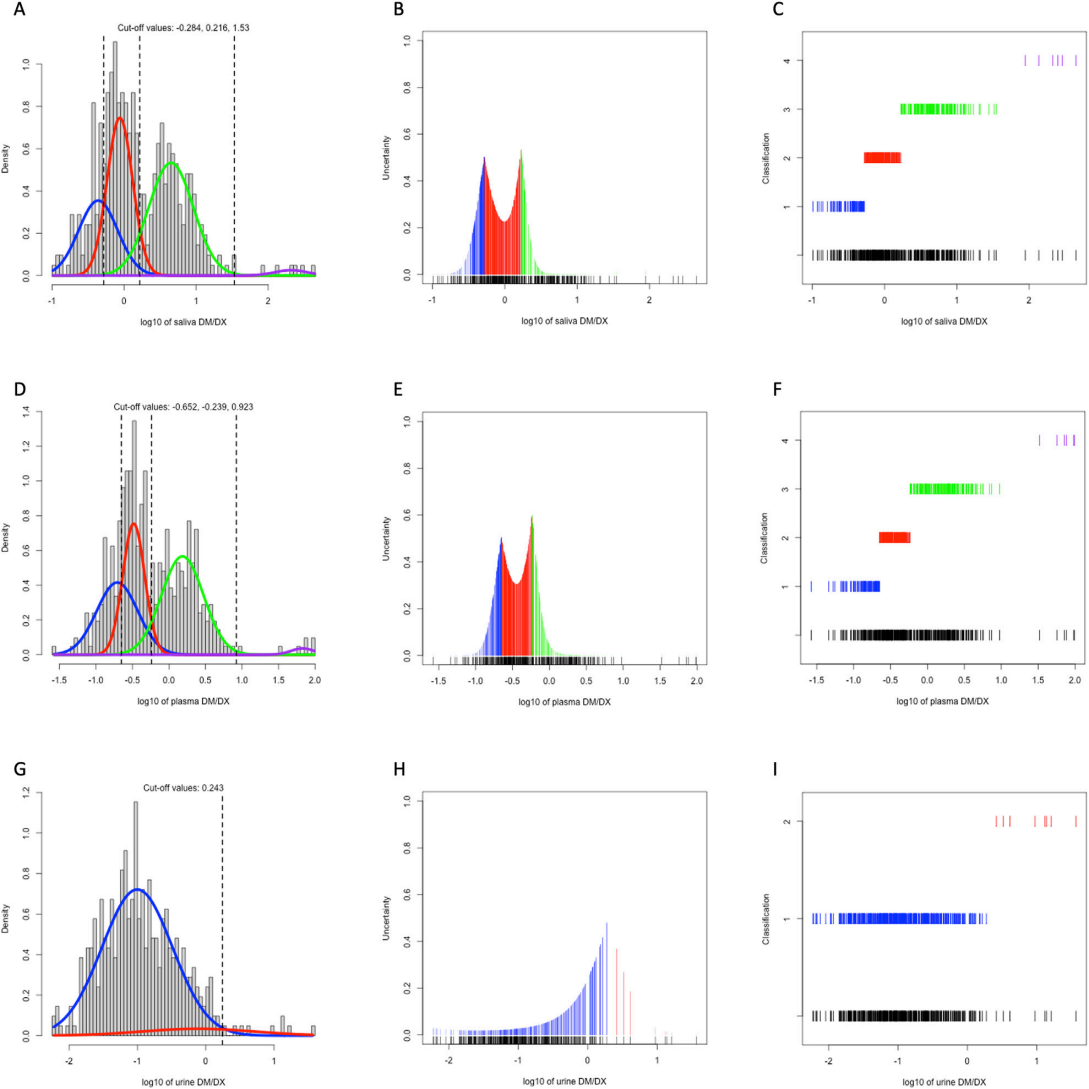
Model-based clustering of the study population based on 3h saliva MR **(A–C)**, 3 h plasma MR **(D–F)**, and 0–3 h urine MR **(G–I)**. The histograms with density curves **(A, D, G)**, the uncertainty plots **(B, E, H)**, and the classification plots **(C, F, I)** were displayed. Colors of all lines represented metabolizer class by AS, with blue for PM, red for IM, green for NM, and purple for UM, respectively.

**FIGURE 2:**
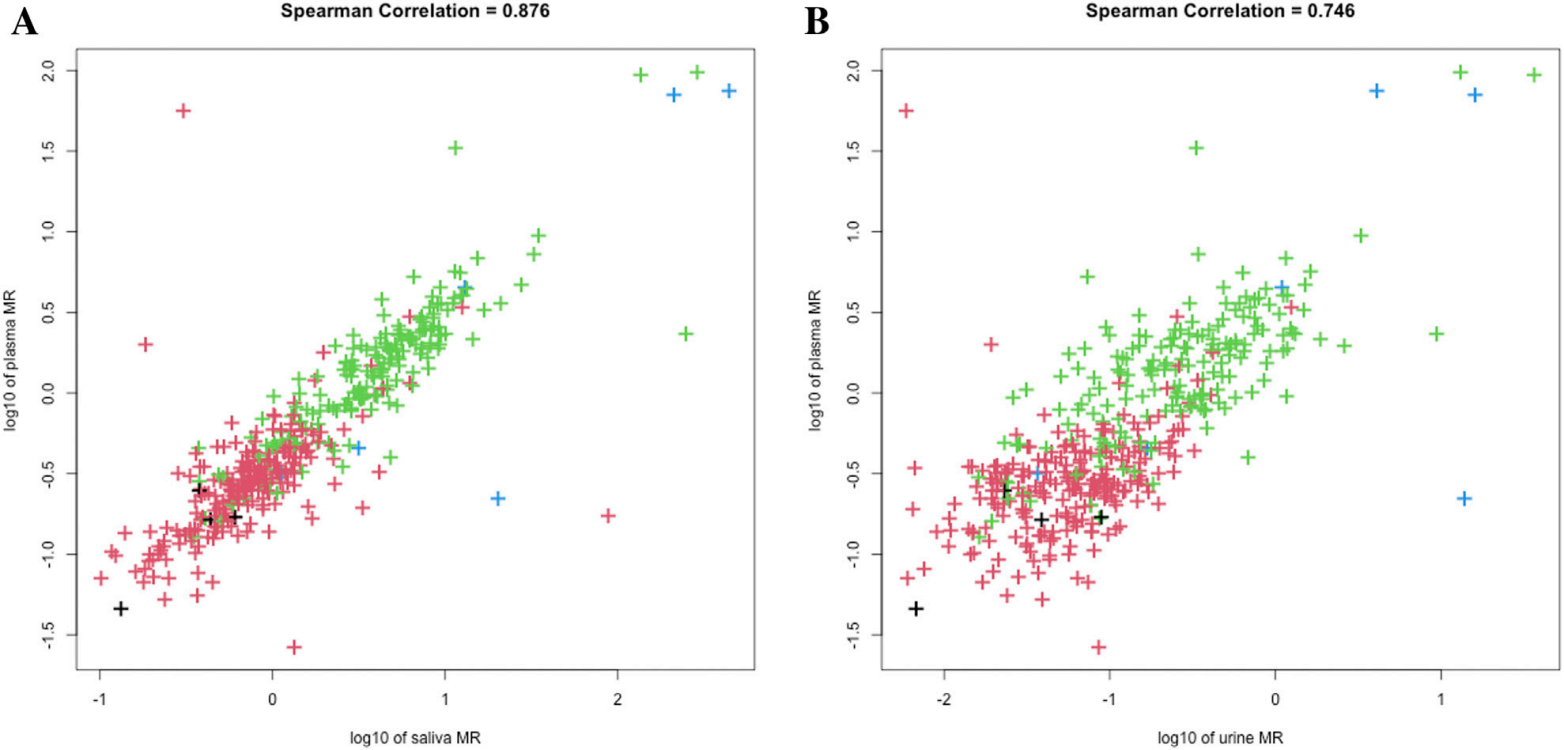
Scatterplots of saliva-plasma MR **(A)** and urine-plasma MR **(B)**. Colors of the dots represented metabolizer class by AS, with black for UM, red for NM, green for IM, and blue for PM, respectively.

We plotted the histogram of log-transformed MR in saliva, plasma, and urine, and performed model-based clustering accordingly (Figure 1). As the classical PM/IM/NM/UM system proposed, both single-point saliva (cutoff values for saliva MR: 0.520, 1.644, 33.884) and single-point plasma (cutoff values for plasma MR: 0.223, 0.577, 8.375) could separate the study population into 4 clusters with fair uncertainty level (Figures 1A–F), and the agreement between saliva- and plasma-based clustering was satisfactory with a Cohen’s kappa of 0.689. In contrast, MR in 0–3 h urine could merely identify the poorest metabolizers from the others with significantly lower kurtosis than saliva and plasma (Figures 1G–I). Moreover, the correlation between plasma and saliva MR (r = 0.876) was closer than that between plasma and urine (r = 0.746), and urine MR seemed more dispersed than saliva MR with a given plasma MR (Figure 2). All these findings indicated that compared with urine MR, saliva MR was a relatively better noninvasive specimen to phenotype *CYP2D6* in the Chinese Han population.

### 3.3 Correlation between 3h saliva MR and AS

**FIGURE 3:**
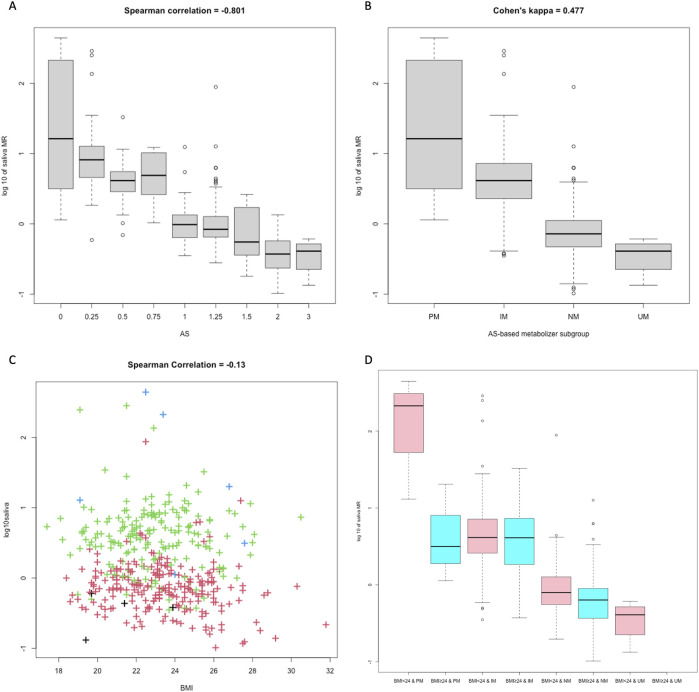
The impact of AS and BMI on 3h saliva MR. Boxplots compared 3h saliva MR between subjects with different AS values **(A)** and different AS-based metabolizer classes **(B)**. The scatterplot showed distribution of 3h saliva MR-BMI **(C)**, and the grouped boxplot **(D)** compared 3h saliva MR of different BMI categories and AS-based metabolizer classes. Colors of the dots represented metabolizer class by AS, with black for UM, red for NM, green for IM, and blue for PM, respectively. In Figure 3D, colors of the graphs distinguish the BMI, as pink for BMI <24, and blue for BMI >24.

A clear monotonic relationship was displayed between AS and single-point saliva MR (r = −0.801, Figure 3A). Fair separation of single-point saliva MR was observed between AS-based UM, NM, and IM metabolizer classes, while the MR distribution was largely intermingled between PM and IM subgroups (Figure 3B). As reported in the confusion matrix (Table 2), the saliva MR-based clustering tended to overstate *CYP2D6* phenotype compared with AS-based metabolizer classes, with the Cohen’s kappa of 0.477 between the two systems.

**TABLE 2:** The confusion matrix comparing saliva MR-based clustering and metabolizer classes by AS, and the AS and saliva MR distribution of different clusters.

	UM	NM	IM	PM	AS	Saliva MR
Cluster 1 (blue)	3	67	7	0	3 (3, 3)	0.41 (0.32, 0.48)
Cluster 2 (red)	1	137	29	1	1.25 (1.25, 2)	0.72 (0.47, 1.11)
Cluster 3 (green)	0	20	142	3	0.5 (0.5, 0.5)	4.12 (2.28, 7.22)
Cluster 4 (purple)	0	1	3	2	0 (0, 0)	16.68 (5.62, 164.82)

### 3.4 Other factors influencing saliva MR

To identify measurable factors other than *CYP2D6* genotype that influence single-point saliva MR, we performed multivariable linear regression for log-transformed 3 h saliva MR (Table 3). When considering BMI, the adjusted *R* ^2^ of the regression model increased significantly compared with the model with AS alone. Overweight subjects with BMI >24 seemed to have lower log-transformed saliva MR compared with others (−0.045 [-0.284, 0.519] vs. 0.132 [-0.147, 0.618], *P* = 0.003 by t-test), and the differences were most apparent in PM subjects (Figures 3C,D).

**TABLE 3:** Multivariable linear regression for log-transformed single-point saliva MR (base 10).

Initial model (adjusted *R* ^2^ = 0.5831)			
Variable	Coefficient	95% confidence interval	*P* value
AS	−0.735	(−0.795, −0.675)	<0.001
Constant	0.970	(0.898, 1.042)	<0.001

Final model (adjusted *R* ^2^ = 0.5875)			
Variable	Coefficient	95% confidence interval	*P* value
AS	−0.728	(−0.788, −0.668)	<0.001
Overweight (BMI >24)	−0.087	(−0.160, −0.014)	0.020
Constant	0.994	(0.919, 1.068)	<0.001

## 4 Discussion

Our study was the first one to demonstrate the performance of single-point saliva for *CYP2D6* phenotyping in a large healthy East Asian population. We demonstrated that *CYP2D6* phenotyping with single-point saliva MR was as good as *CYP2D6* phenotyping with single-point plasma MR for separating different metabolizer subgroups, while the performance of 0–3 h urine MR was not satisfactory. Close correlation was observed between saliva and plasma MR, suggesting that saliva could serve as a satisfactory substitute for plasma in *CYP2D6* phenotyping. The AS system showed fair prediction for single-point saliva-based phenotype, and the prediction could be further improved when taking BMI into consideration.

In order to address interindividual variability in pharmacokinetics in clinical practice, dose adjustment is commonly carried out in two ways: to predict the metabolizer phenotype and prescribe proper dose, or to monitor the drug concentration and titrate the dose (Abdullah-Koolmees et al., 2020). Pharmacogenetic methods, displayed as various AS systems, are expected to define metabolizer subgroups in advance (Jukić et al., 2021). Potato diet-derived solanidine and other metabolic biomarkers have shown promising roles in identifying CYP2D6 PM subjects (Magliocco et al., 2021; Behrle et al., 2022; Wollmann et al., 2023), and linearity was observed between solanidine and risperidone metabolism in certain metabolizer classes (Wollmann et al., 2024). Since not all medical centers have access to the methodology mentioned above, drug concentration monitoring remains irreplaceable up till now, and a non-invasive specimen would be preferrable over plasma.

Saliva should serve as a non-invasive substitute for plasma in most situations other than Sjögren’s syndrome, and should replace urine in patients with kidney dysfunction (Hou et al., 1996). The role of saliva in cytochrome P450 phenotyping and pharmacokinetic monitoring has been confirmed in multiple small-scaled previous studies, and our study is the first to present single-point saliva MR as a suitable measurement for *CYP2D6* phenotype for East Asians (Link et al., 2008; Perera et al., 2011; Chen et al., 2017; Jin et al., 2022). Some drugs like tacrolimus require close monitoring, and drug concentration beyond therapeutic range can lead to detrimental effects including seizure and sinusoidal obstructive syndrome (Xie et al., 2014; Liu et al., 2021). We believe that saliva might be a solution to drug monitoring in pediatric liver transplantation as well as other clinical situations (Yang et al., 2015).

Compared with *CYP2D6* genotyping, single-point saliva MR featured lower cost, lower risk of “genetic identity” exposure, but higher phenotyping uncertainty, which somehow composed a trade-off. There could be a couple of factors underlying the discrepancies between single-point saliva MR-based clustering and *CYP2D6* AS-based metabolizer classification. Although being widely accepted as a *CYP2D6* phenotyping probe, DM is metabolized via CYP3A as well (Tian et al., 2013), which could contribute to the *CYP2D6* phenotype-overstating tendency of saliva MR-based clustering compared with AS. In agreement with a previous study concerning risperidone pharmacokinetics, our study demonstrated the negative impact of BMI on MR of CYP2D6-dependent medication (Paulzen et al., 2016). However, another recent study demonstrated that only CYP3A, not CYP2D6, had its activity significantly correlated with body composition, making things controversial (Krogstad et al., 2021). Further studies with wider BMI distribution should be conducted to analyze the effect of BMI on pharmacokinetics as well as the underlying mechanisms.

Our research had some limitations. The urine collection period was relatively short, which might influence the *CYP2D6* phenotyping performance of urine compared with saliva and plasma. The exclusion of subjects with any morbidity could prevent the widespread application of our data in real clinical settings, and further studies among specific patient groups with clinical endpoints should make up for it. The relatively small number of UM and PM subjects in our cohort might influence the statistical performance of model-based clustering as well as linear regression, and a larger cohort with more UM and PM subjects is expected. Since our phenotyping method, regardless of the sample type, requires chromatography and mass spectrometry equipment that is hard to acquire in most hospitals, a more convenient methodology shall be developed in the future.

In conclusion, our study showed that single-point saliva MR could separate the study population into four metabolizer clusters, and was a convenient and reliable phenotyping method for *CYP2D6* compared with single-point plasma MR and period urine MR. Single-point saliva MR could be predicted with the latest AS system, and the prediction accuracy was improved when taking BMI into account.

## Data Availability

The original contributions presented in the study are included in the article/Supplementary Material, and further inquiries can be directed to the corresponding author.
